# Supplementary material for: Nanoscale assembly processes revealed in the nacroprismatic transition zone of Pinna nobilis mollusc shells
Source: Nat Commun. 2015 Dec 3;6:10097. doi: 10.1038/ncomms10097 (PMC4686775; doi:10.1038/ncomms10097)
Supplement: Supplementary Information — Supplementary Figures 1-10. [file ncomms10097-s1.pdf]

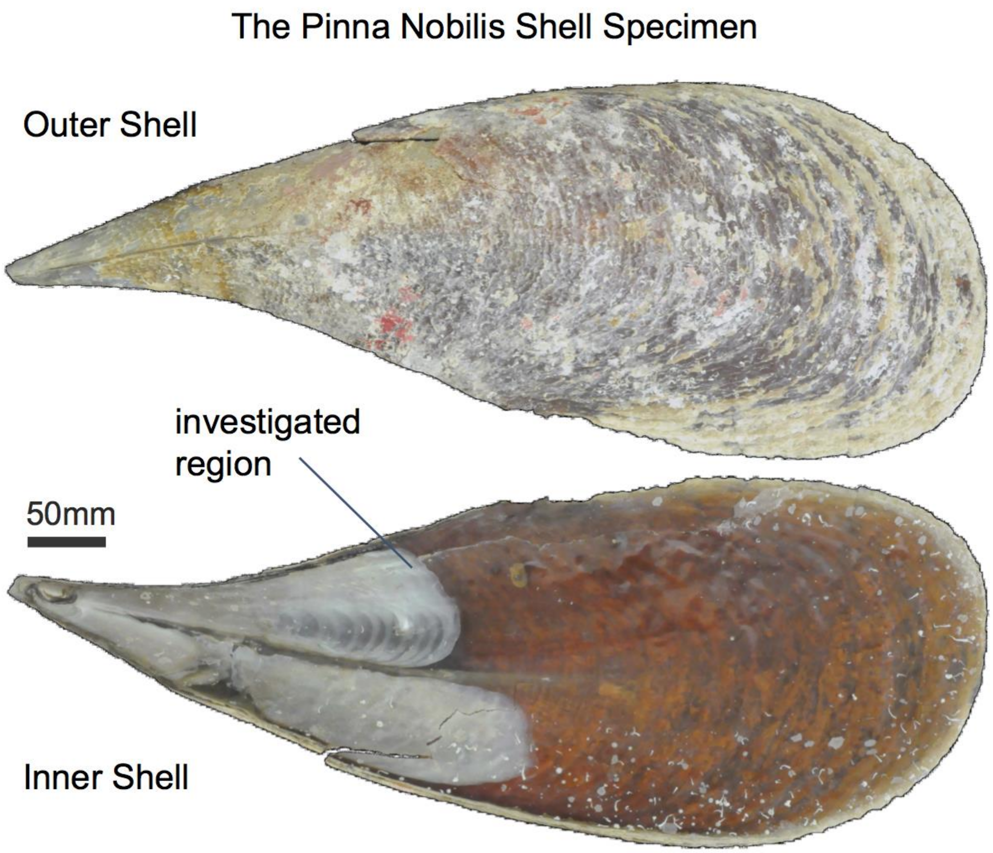

Supplementary Figure 1 | Photographic image of the *Pinna nobilis* shell specimen. The labeled investigated region marks the nacreous area that was characterized by scanning transmission electron microscopy (STEM).

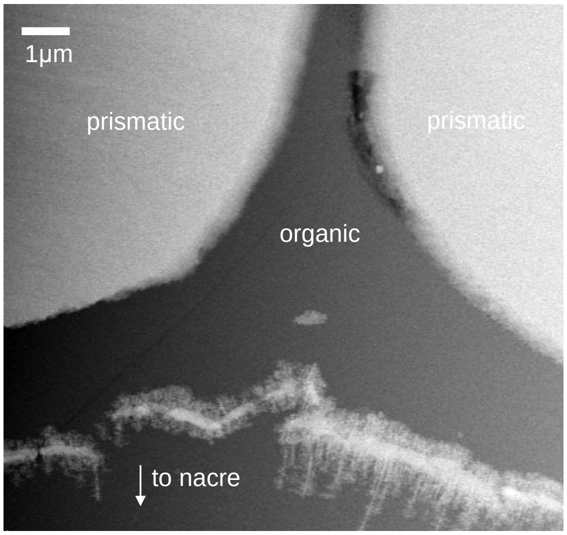

Supplementary Figure 2 | (ADF)-STEM image in cross-section at the termination of the prismatic layer. This termination designates the beginning of the nacroprismatic transition zone. The terminating organic layer has a density indistinguishable from the intra-prismatic organic, appearing as a continuation of the same or similar material.

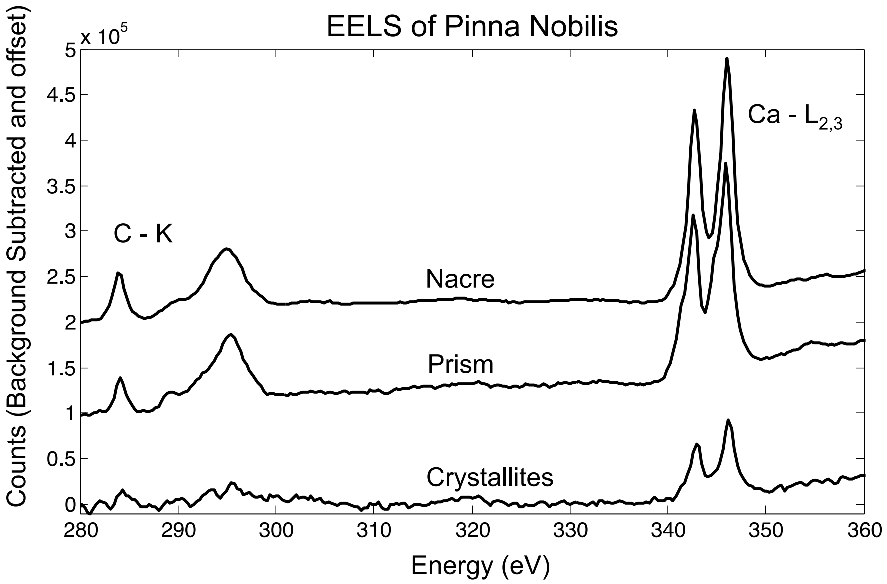

Supplementary Figure 3 | Electron energy loss spectroscopy (EELS) taken in the nacreous, prismatic, and nanocrystal aggregate regions. EELS shows all layers are calcium carbonates. EELS data was collected on a 200 keV FEI Tecnai F20 equipped with a Gatan imaging filter 865-ER.

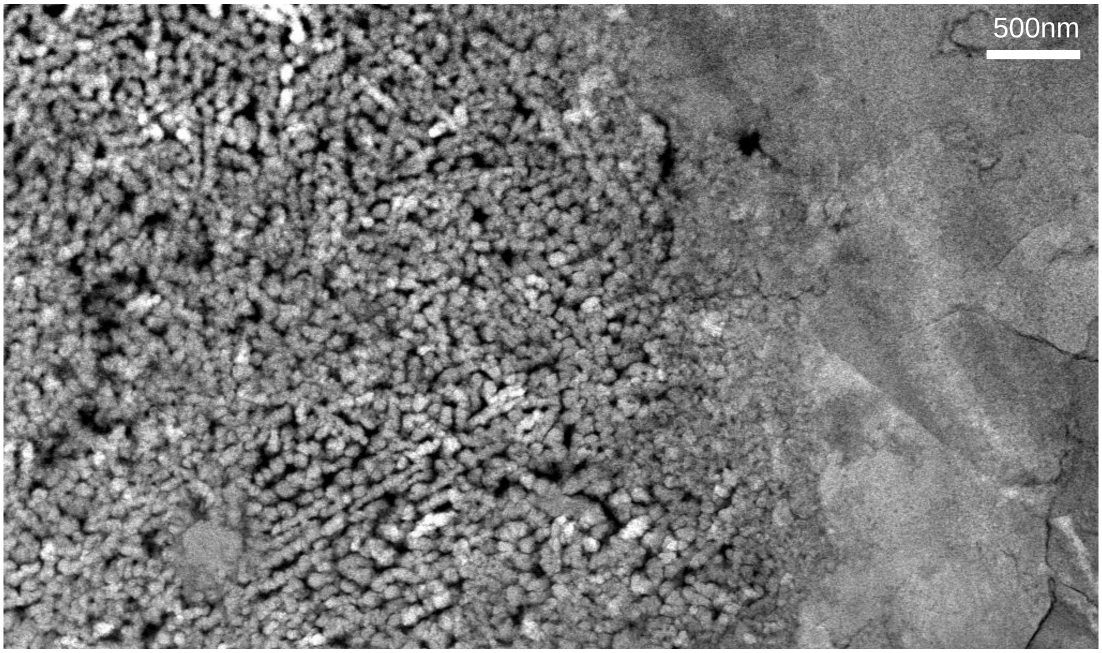

Supplementary Figure 4 | Critical packing of nanocrystallites. Quasi-planar HAADF-STEM image showing the critical packing of nanocrystallites (left) transitioning into the first early-nacre platelets (right).

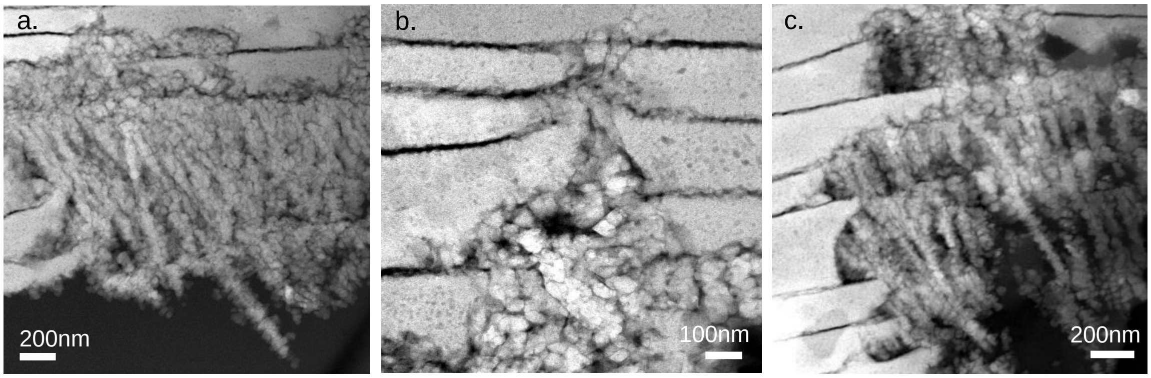

Supplementary Figure 5 | Cross-sectional HAADF-STEM image showing the critical packing of nanocrystallites and the transition into early-nacre platelets. These images show nanocrystal packing at the interface of early-nacre stacking faults.

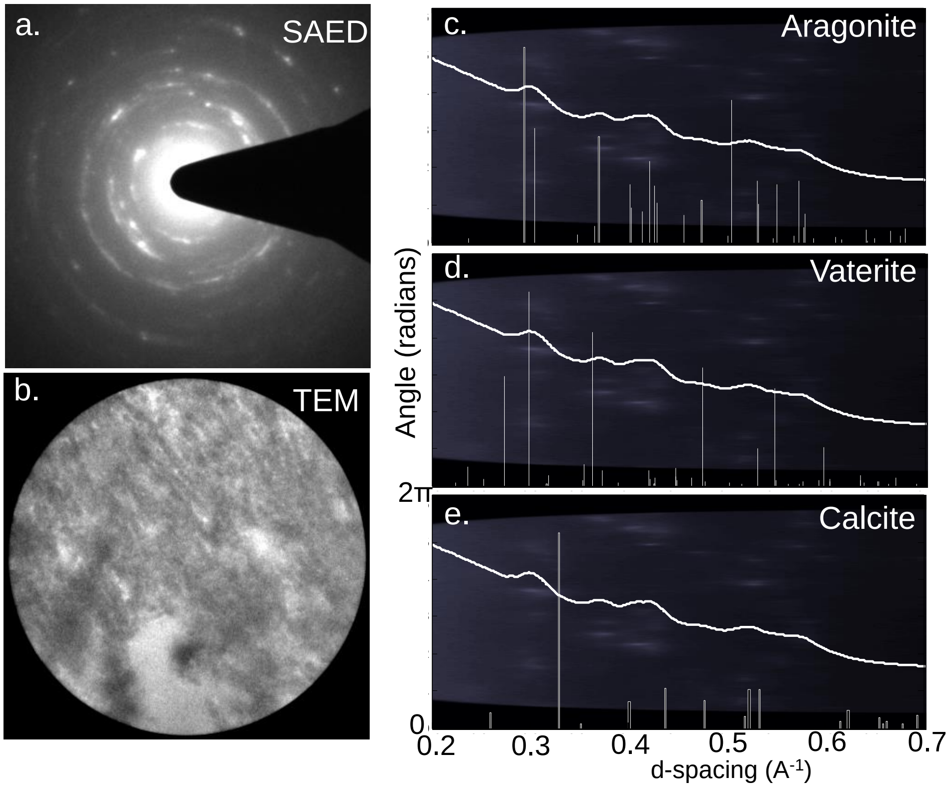

Supplementary Figure 6 | Selected area electron diffraction (SAED) of nanocrystallites in the nanocrystal aggregate formation. (a) SAED pattern of the region shown in (b.) by transmission electron microscopy (TEM). (c-e) the measured radial SAED pattern is compared to the peaks of three calcium carbonate polymorphs: aragonite (c), vaterite (d), and calcite (e). The diffraction pattern of the nanocrystals is consistent with aragonite structure, where measured peaks occur at the expected peaks with aragonite. However, measured peaks occur at places inconsistent with the vaterite and calcite structure. Background of plots (c-e.) is a polar transformation of (a).

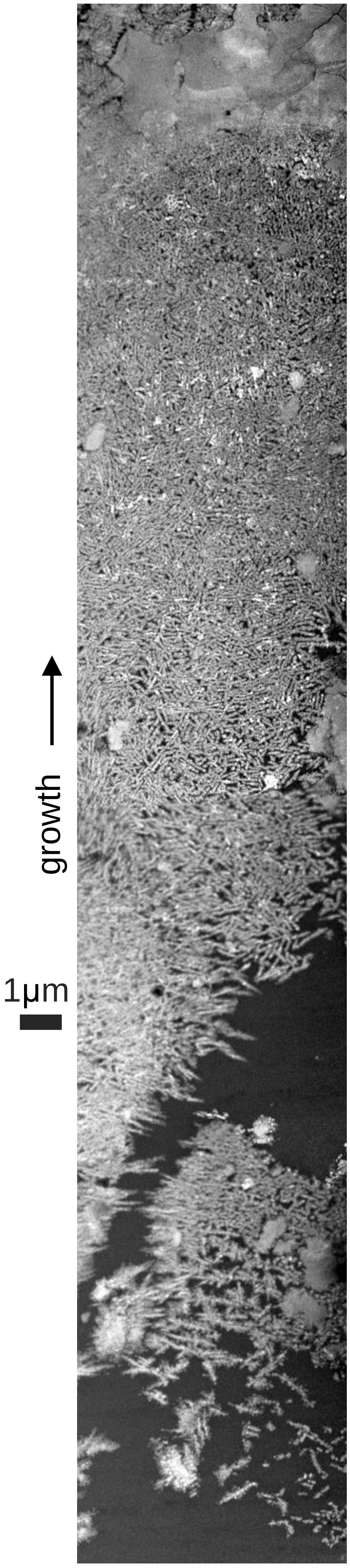

Supplementary Figure 7 | Quasi-planar image at the start of mollusc nacre growth shown at the nanoscale by HAADF-STEM. As time progresses (from bottom to top of Supplementary Figure 7), the nanocrystals aggregate and grow with disordered needle-like geometries. When a critical packing density is reached, continuous early-nacre platelets form – later leading to the familiar large, uniform nacre plates (top). This image is the same data used in Figure 3, only the images are stitched into one continuous composite image.

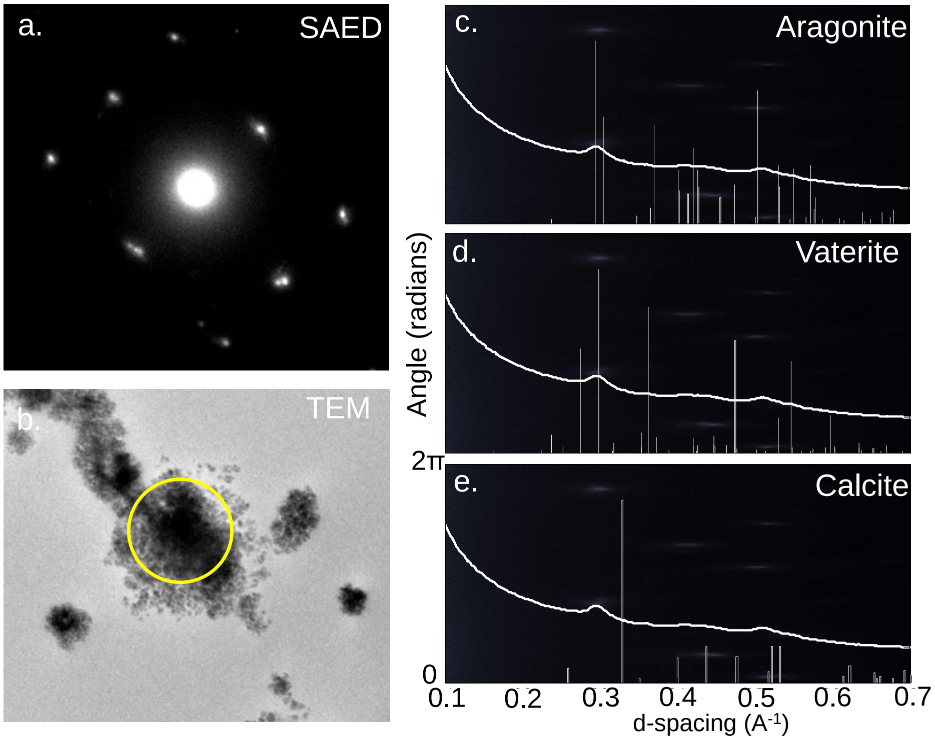

Supplementary Figure 8 | Selected area electron diffraction (SAED) of early $CaCO_3$ formation in the nacroprismatic transition. a) shows the SAED pattern taken from the region shown in b.) by transmission electron microscopy (TEM). c-e) the measured radial SAED pattern is compared to the peaks of three calcium carbonate polymorphs: aragonite (c.), vaterite (d.), and calcite (e.). The diffraction pattern of the nanocrystals is most consistent with aragonite structure, where measured peaks occur at the expected peaks with aragonite. Measured peaks occur at places inconsistent with the vaterite and calcite structure.

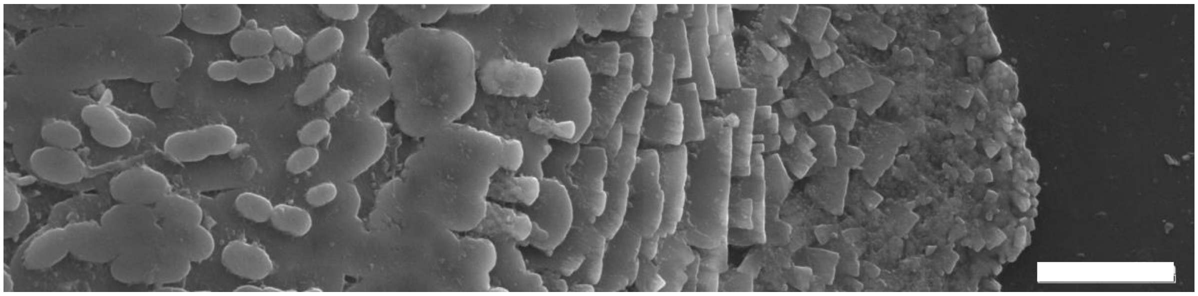

Supplementary Figure 9 | SEM image of early nacre formation as observed on the inward side of the shell. From the green sheet (right), disordered pre-nacre platelets grow layer-by-layer, increasing progressively in size, and with more order. Eventually the familiar ordered nacre plates are formed (left). Scale bar 10 μm.

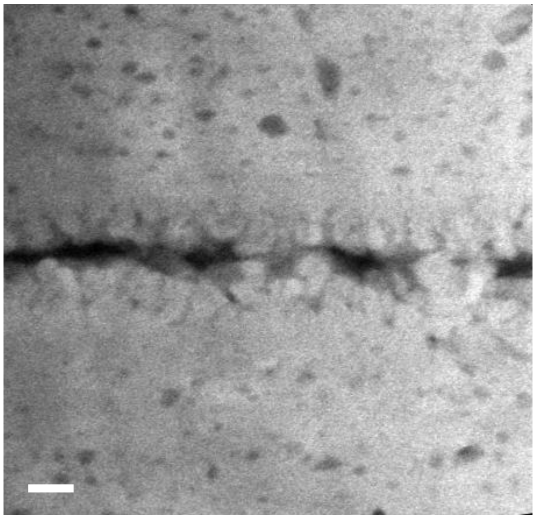

Supplementary Figure 10 | Inorganic (Checa) bridges connect two nacre layers separated by an organic bridge. Subnanometer detail of the interface is imaged using HAADF-STEM. Scale bar 20 nm.
